# Misclassification of current status data

**DOI:** 10.1007/s10985-010-9154-0

**Published:** 2010-02-16

**Authors:** Karen McKeown, Nicholas P. Jewell

**Affiliations:** Division of Biostatistics, School of Public Health, University of California, 101 Haviland Hall MC 7358, Berkeley, CA 94720, USA

**Keywords:** Current status data, Misclassification

## Abstract

We describe a simple method for nonparametric estimation of a distribution function based on current status data where observations of current status information are subject to misclassification. Nonparametric maximum likelihood techniques lead to use of a straightforward set of adjustments to the familiar pool-adjacent-violators estimator used when misclassification is assumed absent. The methods consider alternative misclassification models and are extended to regression models for the underlying survival time. The ideas are motivated by and applied to an example on human papilloma virus (HPV) infection status of a sample of women examined in San Francisco.

## 1 Introduction

Current status data provides information on the survival status of individuals at various times rather than standard observation, possibly right-censored, of failure times. Considerable attention has been given to estimation of a survival function based on such data, and estimation of regression coefficients from a variety of standard models. Earliest work was motivated by applications in demography (Diamond et al. 1986) and epidemiology (Becker 1989), followed by carcinogenicity studies, partner studies of Human Immunodeficiency Virus (HIV) transmission (Shiboski and Jewell 1992), age-incidence estimation, and assessment of environmental exposures (Keiding 1991). Nonparametric estimation in the single-sample setting is based on the well-known pool-adjacent-violators algorithm of Ayer et al. (1955). Regression analyses have largely employed techniques from generalized linear models for the current status outcome and variants of generalized additive models (Shiboski 1998). A brief review and description of some current open problems can be found in Jewell and van der Laan (2004).

In many of these applications, ascertainment of an individual’s current status is based on a screening test which may not have perfect sensitivity and specificity. For example, tests for the infection status of a viral disease like HIV or HPV are designed to detect antibodies and may be subject to error particularly when a test is performed soon after infection. Detection of the existence of uterine fibroids through ultrasounds (Young et al. 2008) is known to be subject to error. When current status is measured through a survey instrument, such as studies of the age at onset of menopause (Grummer-Straun 1993; Jewell et al. 2003), there is potential for misclassification particularly close to the (unobserved) event time, menopause in this specific example.

We extend the nonparametric maximum likelihood estimator of the distribution function underlying current status data when there is no misclassification to allow for time-independent misclassification of both apparent “survivors” and “failures” with known misclassification rates. Calculation of the proposed estimator uses a simple modification of the pool-adjacent-violators algorithm. Asymptotic properties therefore follow straightforwardly. We consider the implication of misclassification rates that vary over time, in particular when misclassification only occurs in a known time window surrounding the underlying failure event. We also consider regression models for current status data subject to misclassification, using the ideas for binary generalized linear models with outcome subject to misclassification (Neuhaus 1999).

## 2 Nonparametric estimation of a single distribution function

We assume the standard data structure for current status data with the following notation. Let *T* be the survival time random variable of interest with distribution function *F*, with the monitoring time denoted by the random variable *C*. As usual, we assume that *C* is independent of *T*; in some examples, *C* is non-random. In either case, we focus directly on the conditional likelihood, given *C*. For convenience we describe the random monitoring time scenario, where current status observation refers to a sampling scheme where *n* i.i.d. observations are collected on the random variable (*Y*, *C*) where *Y* = *I*(*T* ≤ *C*).

Motivated by the examples discussed in the introduction we now consider the possibility that the random variable *Y* is observed with error. We focus primarily on the following constant misclassification model although we discuss alternative error models in Sect. 2.3. Assume that instead of observing *Y* we observe the random variable Δ where

P(Δ=1∣Y=1)=α P(Δ=0∣Y=0)=β.

The observed data is thus *n* i.i.d. copies of (Δ, *C*).

We assume that the true classification probabilities *α*, *β* > 0.5, and are the same for each individual and do not depend on the monitoring time. Let *C*_*i*_ be the *i*th order statistic of *C*_1_, *C*_2_, …, *C*_*n*_ and let *δ*_*i*_ be the observed value of Δ. Given that the monitoring time *C* is independent of survival time *T*, and that Δ is independent of (*C*, *T*), under this misclassification model, the (conditional) likelihood function is given by:

(1) $$\prod_{i=1}^{n}{\left[P\left(\Delta_{i}=1|c_{i}\right)\right]}^{\delta_{i}}{\left[P\left(\Delta_{i}=0|c_{i}\right)\right]}^{1-\delta_{i}},$$

where *c*_*i*_ is the observed value of *C*_*i*_ and

$$P\left(\Delta_{i}=1|c_{i}\right)=P\left(\Delta_{i}=1|y_{i}=1,c_{i}\right)P\left(y_{i}=1|c_{i}\right)\;+P\left(\Delta_{i}=1|y_{i}=0,c_{i}\right)P\left(y_{i}=0|c_{i}\right)\;=(\alpha -1+\beta )F\left(c_{i}\right)+1-\beta ,$$

and

$$P\left(\Delta_{i}=0|c_{i}\right)=P\left(\Delta_{i}=0|y_{i}=0,c_{i}\right)P\left(y_{i}=0|c_{i}\right)\;+P\left(\Delta_{i}=0|y_{i}=1,c_{i}\right)P\left(y_{i}=1|c_{i}\right)\;=\beta -(\alpha -1+\beta )F\left(c_{i}\right).$$

For ease of notation let *γ* = *α* + *β* − 1 > 0. Then the (conditional) likelihood function allowing for misclassification in the response variable can be written as;

$$\prod_{i=1}^{n}{\left[\gamma F\left(c_{i}\right)+(1-\beta )\right]}^{\delta_{i}}{\left[\beta -\gamma F\left(c_{i}\right)\right]}^{1-\delta_{i}},$$

with corresponding log-likelihood:

$$\sum_{i=1}^{n}\delta_{i}\text{log}\left(\gamma F\left(c_{i}\right)+(1-\beta )\right)+\sum_{i=1}^{n}\left(1-\delta_{i}\right)\text{log}\left(\beta -\gamma F\left(c_{i}\right)\right).$$

Writing *G*(*c*_*i*_) *≡ γF*(*c*_*i*_) + (1 − *β*), then the nonparametric maximum likelihood estimate of the distribution function when the current status outcomes are subject to misclassification can be found by obtaining a vector $\tilde{z}=\left(z_{1}=\hat{G}\left(c_{1}\right),\ldots ,z_{n}=\hat{G}\left(c_{n}\right)\right)\in R^{n}$ maximizing

(2) $$\phi \left(G\left(c_{i}\right)\right)=\sum_{i=1}^{n}\delta_{i}\;\text{log}\left(G\left(c_{i}\right)\right)+\sum_{i=1}^{n}\left(1-\delta_{i}\right)\;\text{log}\left(1-G\left(c_{i}\right)\right)$$

under the constraint

(3) $$1-\beta \leq G\left(c_{1}\right)\leq G\left(c_{2}\right)\leq \cdots \leq G\left(c_{n}\right)\leq \alpha .$$

Note that *G* is itself a distribution function.

Claim *The identity* $z_{m}=\text{min}\left(\text{max}\left({\hat{z}}_{m},1-\beta \right),\alpha \right)$, *m* = 1, …, *n, defines the unique vector*, $\tilde{z}=\left(z_{1},z_{2},\ldots ,z_{n}\right)\in {\mathbb{R}}^{n}$ *maximizing* (2) *under constraint* (3)*, with G*(*c*_*i*_) *replaced by z*_*i*_*, where*

$${\hat{z}}_{m}=\underset{i\leq m}{\text{max}}\;\underset{k\geq m}{\text{min}}\;\frac{\sum_{i\leq j\leq k}\delta_{i}}{k-i+1}$$

*is the unconstrained nonparametric maximum likelihood estimate* (*NPMLE*) *of the distribution function G based on the likelihood* (2) *but with no additional constraint* (3).

Note that the vector $\left\{{\hat{z}}_{m}\;:\;m\;=\;1,\;\ldots \;,n\right\}$ can be computed using the standard pool-adjacent-violators algorithm, originally described by Ayer et al. (1955) and characterised by Barlow et al. (1972) and Groeneboom and Wellner (1992) in terms of convex minorants. The vector {*z*_*m*_} modifies any value of $\left\{{\hat{z}}_{m}\right\}$ less than 1 − *β* to equal 1 − *β*, and similarly modifies any value of $\left\{{\hat{z}}_{m}\right\}$ greater than *α* to equal *α*. The NPMLE of *F* at a monitoring time *c*_*i*_ then follows using the relationship $\hat{F}\left(c_{i}\right)=\left[\hat{G}\left(c_{i}\right)-1+\beta \right]/\gamma$.

*Proof of Claim* First note that, if *δ*_*i*_ = 0 for *i* = 1, 2, …, *k*, then maximizing (2) requires the second term to be as large as possible, in which case, we set *z*_1_, *z*_2_, …, *z*_*k*_ = 1 − *β* without affecting the maximization problem over the remaining *z*_*k+*1_, …, *z*_*n*_. Similarly, if *δ*_*i*_ = 1 for *j* ≤ *i* ≤ *n*, then to maximize (2) we make the first term as large as possible, setting *z*_*j*_*, z*_*j+*1_, …, *z*_*n*_ = *α*.

Suppose there exists at least one *δ*_*i*_ = 1 followed by a *δ*_*j*_ = 0, for some *j* > *i* (otherwise we are done).

Let *k*_0_ be the smallest index *i* such that *δ*_*i*_ = 1, and let *k*_1_ be the smallest index *k* ≥ *k*_0_ such that

$$\underset{i\leq m}{\text{max}}\;\underset{k\geq m}{\text{min}}\;\frac{\sum_{i\leq j\leq k}\delta_{i}}{k-i+1}\geq 1-\beta .$$

Analogously, let *m*_0_ be the largest index *k* ≥ *k*_1_ such that

$$\underset{i\leq m}{\text{max}}\;\underset{k\geq m}{\text{min}}\;\frac{\sum_{i\leq j\leq k}\delta_{i}}{k-i+1}\leq \alpha ,$$

with *m*_1_ being the largest index *i* such that *δ*_*i*_ = 0.

**Fig. 1:**
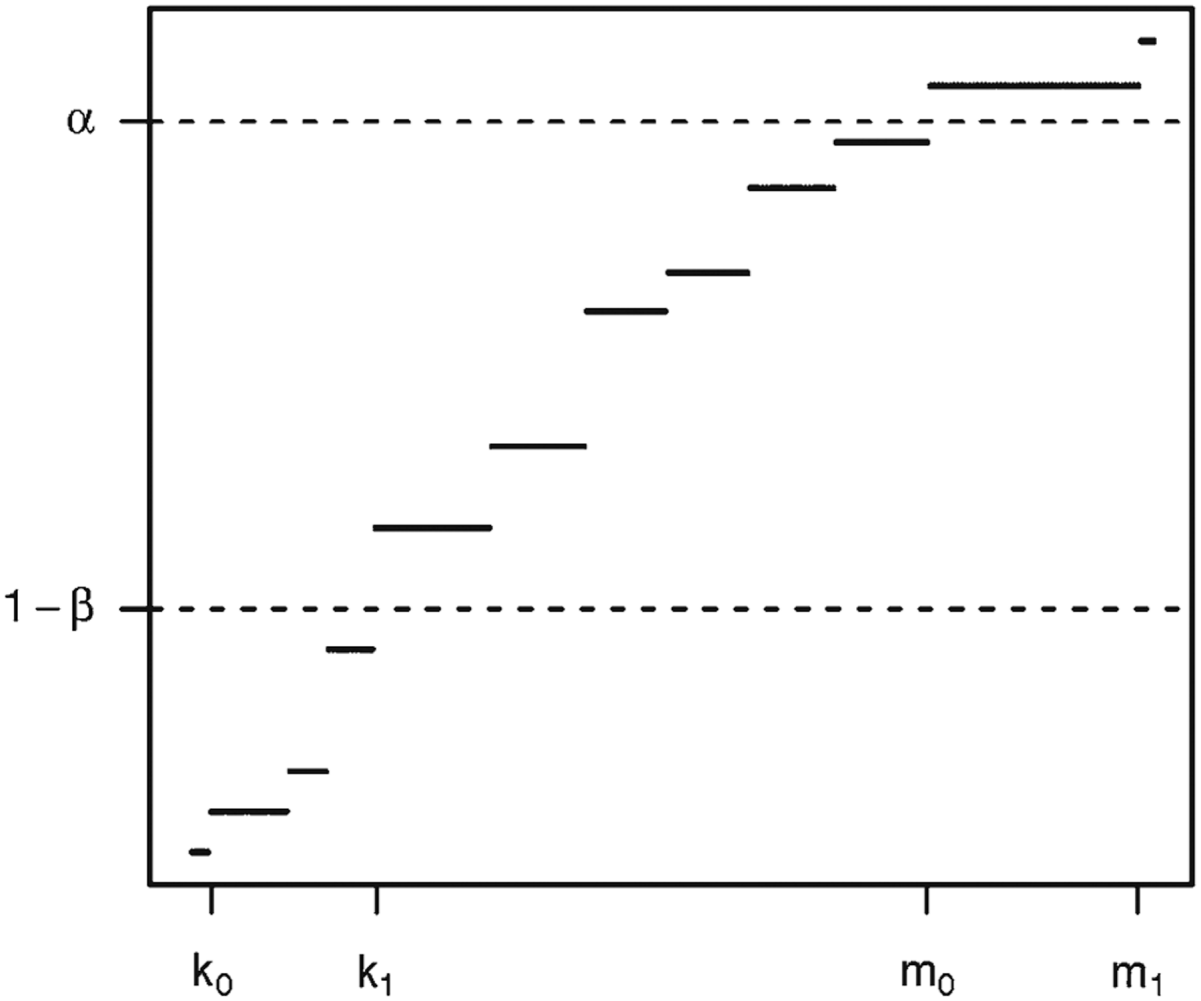
Hypothetical unconstrained NPMLE with the positions of hypothetical *α* and 1 − *β* shown on the vertical axis and the positions of *k*_0_*, k*_1_*, m*_0_ and *m*_1_ shown on the horizontal axis

Thus, *k*_0_ and *m*_1_ represent the index of the first *δ*_*i*_ = 1 and the last *δ*_*j*_ = 0 respectively, where *j* > *i*. Also, *k*_1_ − 1 is the smallest index for which the unconstrained NPMLE does not fall below 1 − *β*, and *m*_0_ is the largest index for which the unconstrained NPMLE does not go above *α*. Figure 1 shows the positioning of such indices as they would appear in terms of a hypothetical unconstrained NPMLE of a distribution function. The dashed lines are positioned at 1 − *β* and *α*, between which the constrained NPMLE must lie. □

Using these definitions, the claim can be written as;

- For all indices *m* < *k*_1_*, z*_*m*_ = 1 − *β*
- For all indices *m* > *m*_0_*, z*_*m*_ = *α*
- For all indices *k*_1_ ≤ *m* ≤ *m*_0_; $z_{m}={\text{max}}_{i\leq m}\;{\text{min}}_{k\geq m}\frac{\sum_{i\leq j\leq k}\delta_{i}}{k-i+1}$, the unconstrained NPMLE.

We prove the claim by establishing each statement separately. First, we show that for all indices *m* < *k*_1_*, z*_*m*_ = 1 − *β* maximizes the relevant terms in the likelihood (2), subject to the constraint (3) without affecting the optimization function, or constraint, based on *z*_*i*_ for other indices. Consider indices *i* for *k*_0_ ≤ *i* < *k*_1_. Suppose the values of *z*_*i*_ over this range of indices take values that are increasing and, necessarily, ≥1 − *β*. Consider the largest of these indices (just to the “left” of *k*_1_) where the proposed maximizer values of *z*_*i*_ assume the value 1 − *β* + *ϵ* where *ϵ* > 0. It does not matter here whether *z*_*i*_ assumes this value at one or over a set, *S* of consecutive indices. Assume that amongst the set of indices, *S*, there are *p* indices *i* where *δ*_*i*_ = 1 and *q* indices where *δ*_*i*_ = 0. The contribution to the likelihood (2) over this set of indices is therefore *p* log(1 − *β* + *ϵ*) + *q* log(*β* − *ϵ*) ≡ *h*(*ϵ*), say. The derivative of this function is *h*′(*ϵ*) = [*p*/(1 − *β* + *ϵ*)] − [*q*/(*β* − *ϵ*)]. Now, by the definition of *k*_1_ relative to the definition of the unconstrained NPMLE, it follows that *p*/(*p* + *q*) < 1 − *β* that in turn implies that *q*/*p* > *β*/(1 − *β*). Since *ϵ* > 0, *β*/(1 − *β*) > (*β* − *ϵ*)/(1 − *β* + *ϵ*), and it then follows that *h*′(*ϵ*) < 0 so that *h* is decreasing in *ϵ*. Thus without changing the optimization problem in terms of the other indices and constraints, we can increase the likelihood by lowering the value of the proposed *z*_*i*_ to the next lower value (to the right) where *z*_*j*_ = 1 − *β* + *λ* where 0 < *λ* < *ϵ*. However, we can now repeat the same argument in terms of *λ*, and thus we keep lowering the relevant *z*_*i*_’s until they all equal 1 − *β*. This proves (A). An identical argument also establishes (B). The statement (C) follows since $z_{m}={\text{max}}_{i\leq m}\;{\text{min}}_{k\geq m}\frac{\sum_{i\leq j\leq k}\delta_{i}}{k-i+1}$ is already the unconstrained NMPLE and meets the constraints (3) by definition of *k*_1_ and *m*_0_. The claim is thus proven.

### 2.1 Pointwise confidence intervals for the NPMLE

There is by now a growing literature on the non-standard asymptotic properties of the standard NPMLE of *F* for current status data with no misclassification. There is a slower rate of convergence (*n*^1/3^ as opposed to the familiar $\sqrt{n}$ rate), and the limit distribution is not Gaussian (Groeneboom and Wellner 1992). We conjecture straightforward extensions of these results for the NPMLE for misclassified current status data. Thus it is not appropriate to focus on the (asymptotic) variance of the NPMLE based on any form of current status data as a step towards confidence interval construction. For pointwise confidence intervals for *F*, various approaches have been developed for standard current status data (Banerjee and Wellner 2005). Suggested techniques include the likelihood-ratio method (Banerjee and Wellner 2001), an approach that can presumably also be adapted to allow for misclassification.

In general, the standard bootstrap yields inconsistent estimates of pointwise confidence intervals whether data is sampled with replacement from the original data or generated from the NPMLE estimator (Sen et al. 2010). As a modification, a smoothed version of the bootstrap is appropriate, as is the *m* out of *n* bootstrap (Politis et al. 1999). Practically, this procedure necessarily involves choice of the ‘block’ size *m*. Asymptotically, *m* must be chosen so that *m →* ∞ and *m*/*n →* 0 as *n →* ∞ although these requirements provide little guidance for a finite sample size. Banerjee and Wellner (2005) suggest an intricate procedure for choice of *m*, based itself on bootstrapping. The method can be adapted to provide symmetric confidence intervals as these often perform better in finite samples. Banerjee and Wellner (2005) provide further implementation details. For current status data with misclassification, illustrative calculations of symmetric confidence intervals using the *m* out of *n* bootstrap are provided in Sect. 2.2.

### 2.2 Illustration and data example

**Fig. 2:**
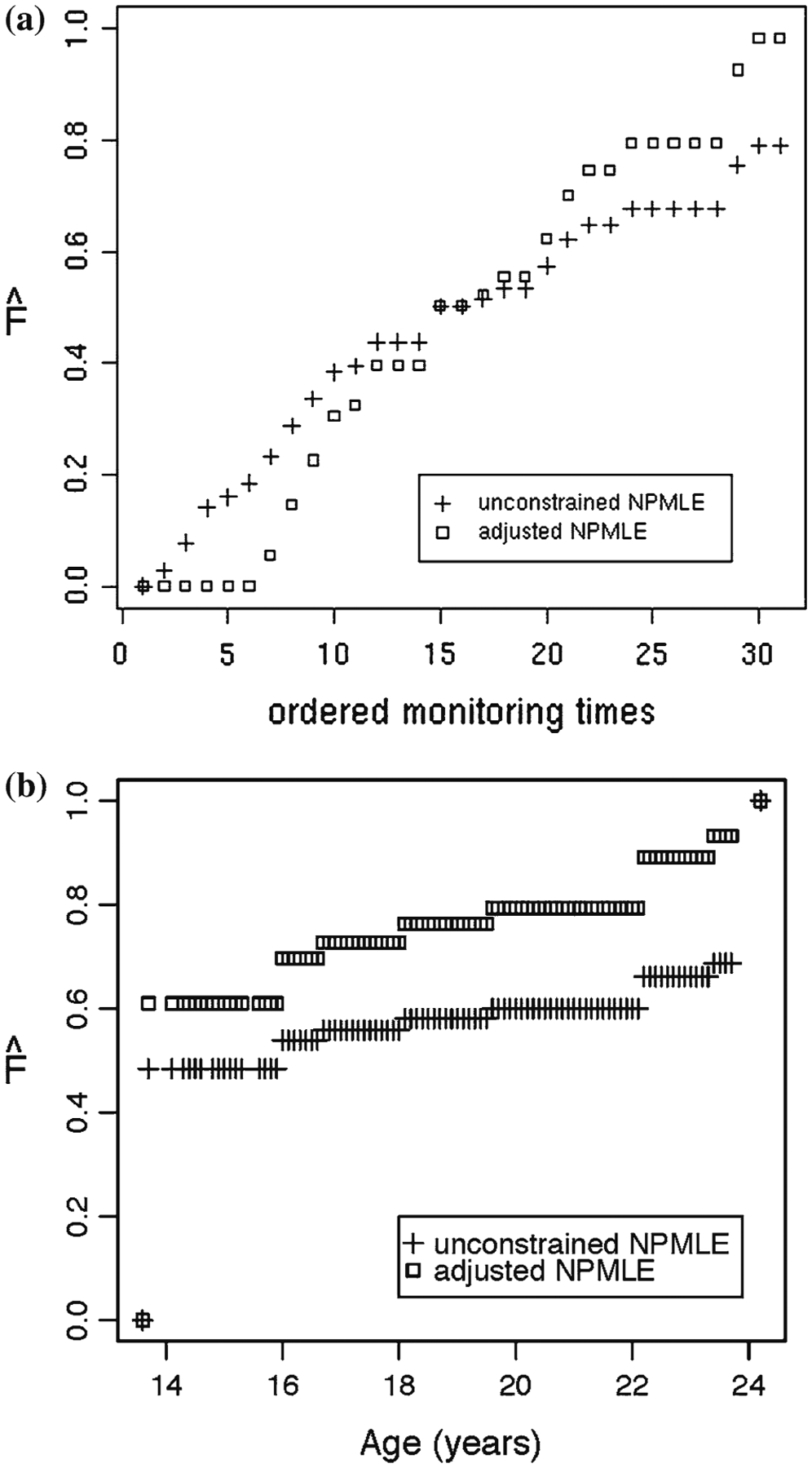
**a** Hypothetical data (*α* = 0.8, *β* = 0.8). **b** HPV data (*α* = 0.73, *β* = 0.9). Estimated cumulative distribution functions for hypothetical data (*F* assumed Exponential with mean 2) and the HPV data. Both the unconstrained NPMLE obtained through the pool-adjacent-violators algorithm and the proposed adjusted NPMLE are presented

First, Fig. 2a illustrates the unconstrained NPMLE and the NPMLE adjusted for misclassification for a hypothetical data set with sample size *n* = 500 generated from an exponential distribution, *F*, with mean 2. The monitoring times were selected at random from a uniform distribution on a set of discrete time values ranging from 0 to 3 at equal increments of 0.1. The classification rates used in generating the data were *α* = *β* = 0.8, and these values were assumed known in calculating the adjusted NPMLE. Note that, with *α* = *β*, the two estimators cross at $\hat{F}=0.5$, the estimated median time to occurrence; for time points below this value, the adjusted estimate of *F* is shifted downwards from the naive estimator as misclassifications are accounted for, and similarly shifted upwards at values of time above the estimated median.

Current status data on human papilloma virus (HPV) infection among women motivate and illustrate this work. The study consisted of 827 women aged 13.5–24.2 years examined in San Francisco (Moscicki et al. 1998). The data contained a binary indicator of whether a woman has HPV infection at the time of the survey (*Y*) and her age at screening (*C*). Covariates included indicators of current smoking status and past infection with any other sexually transmitted disease (STD). For more information about the dataset see Neuhaus (1999) where it was assumed that HPV testing approach enjoyed (correct) classification rates of *α* = 0.8 and *β* = 0.9. We note that more advanced screening instruments for HPV are now available.

In this example we first need to consider the definition of the underlying failure time since HPV infection can sometimes go into remission in the sense that negative tests can plausibly follow an earlier true positive test. Here we define *T* to be age at *first* HPV infection as distinct from the cross-sectional prevalence interpretation used by Neuhaus (1999). In this case, we allow for additional misclassification of apparently negative screens as such individuals may previously have been infected. We assume that such misclassification applies to 10% of negative screening results. This additional misclassification reduces the value of *α* to 0.73 [*α* = *P*(Δ = 1|*Y* = 1) = *P*(Δ = 1|*Z* = 1*, Y* = 1)*P*(*Z* = 1|*Y* = 1) + *P*(Δ = 1| *Z* = 0*, Y* = 1)*P*(*Z* = 0|*Y* = 1), where *Z* = 1 if individual has antibodies]. Based on the HPV data, Fig. 2b displays both the unconstrained NPMLE estimate of age at onset of HPV, and the NPMLE adjusted for misclassification with the assumed values *α* = 0.73 and *β* = 0.9, which allows for the additional misclassification discussed above. With these unequal classification probabilities, the two curves cross at $\hat{F}=0.270$, with the adjusted NPMLE shifted appropriately higher for higher ages. We do not see the shift downwards for lower ages since the first jump of the unconstrained NPMLE is to a value higher than 0.270.

95% symmetric confidence intervals were calculated for the adjusted (*α* = 0.73, *β* = 0.9) NPMLE using the *m* out of *n* bootstrap noted in Sect. 2.1. Table 1 provides the results of such calculations at three monitoring times for various choices of *m* ranging from 9 to 423. These values of the block sizes, *m*, were chosen based on the block sizes implemented in the simulations of Politis et al. (1999). The results are quite stable across these choices except perhaps at 15.3 years. It it noteworthy that 15.3 years is close to the smallest monitoring times; in fact, it is at the first jump of the estimator. In Table 1 slightly more variability is suggested for the central values of *m*; however, overall the results provide a clear, consistent and useful assessment of variability.

### 2.3 Misclassification that varies over time

We now consider an extension of the simple constant (i.e. time independent) misclassification model to allow for the misclassification rates to vary over time. In particular, we consider the situation where one or both misclassifications occur only when the monitoring time is close to the time of the true event occurrence. This is natural for screening tests where accuracy may be essentially perfect far from the event time on either side but where misclassification is likely when screening is administered just before or after the event of interest. For example, with current status assessment of menopause, misclassification is unlikely for a woman of age 30 or 65, but may be plausible at age 50. In diagnosing HPV infection, the probability of a false negative possibly decreases with time since infection.

We examine the simple extension where misclassification occurs only in a time window surrounding the true failure event *T* given by [*T* − *A*, *T* + *A*]. Within this interval we assume that the classification rates *α*, *β* > 0.5 are known, that perfect classification occurs at screening times outside the window, and that the value *A* is also known. Using these assumptions we obtain the following log-likelihood;

(4) $$\sum_{i=1}^{n}\delta_{i}\;\text{log}\left((1-\alpha )F\left(c_{i}-A\right)+(\alpha -(1-\beta ))F\left(c_{i}\right)+(1-\beta )F\left(c_{i}+A\right)\right)\;+\sum_{i=1}^{n}\left(1-\delta_{i}\right)\;\text{log}(1-((1-\alpha )F\left(c_{i}-A\right)+(\alpha -(1-\beta ))F\left(c_{i}\right)\;+(1-\beta )F\left(c_{i}+A\right))).$$

Note, when *A* = 0 and *A* = ∞, (4) reduces to the conditional log-likelihood of the unconstrained NPMLE and the conditional log-likelihood with constant misclassification rates, respectively. The more complex conditional log-likelihood is still of the form given in (2) if we define a distribution function G*(*c*_*i*_) *≡* (1 − *α*)*F*(*c*_*i*_ − *A*) + (*α* + *β* − 1)*F*(*c*_*i*_) + (1 − *β*)*F*(*c*_*i* +_ *A*). However, finding the NPMLE of G* is complicated here by the fact that the constraint on G* (as *c →* 0) depends on the unknown value *F*(*A*). In addition, even if a reasonable estimator of G* is determined, it is not generally possible to solve for *F* in terms of G*. This identifiability issue is most easily seen when there is but a single monitoring time, *C*; in this situation, only G*(*C*) is identifiable from the data and differing values of *F*(*C*) (and *F*(*C* − *A*) and *F*(*C* + *A*)) are compatible with any given value for G*(*C*). However, this does not address identifiability when the observed monitoring times cover a much broader range. In the latter situation, it is possible to make bias modifications to either the unconstrained or adjusted NPMLE to address an incorrect misclassification assumption. This allows the proposed and unconstrained estimators to accommodate a different window of misclassification than assumed by either estimator; the approach is formally introduced, discussed and evaluated via simulations in the next subsection.

### 2.4 Time-varying misclassification: simulations

We carried out a set of simulations to examine the implications of misclassification rates that vary over time. Data sets of unobserved event times, of sample size 500, were generated from an Exponential distribution, *F*, with mean 2. Current status observations were then created based on monitoring times selected at random from a Uniform distribution on a set of discrete time values ranging from 0 to 3 at equal increments of 0.2. Finally, the current status data were (mis)classified with classification probabilities of *α* = *β* = 0.8 if and only if *|C*_*i*_ − *T*_*i*_*|* ≤ *A* in order to obtain the data set used in estimation. Outside this window the current status responses were observed without error. A variety of values of *A* were examined including *A* = 0 (no misclassification) and *A* = ∞ (constant misclassification).

For each data set, estimates of $\hat{F}$ were obtained according to both the unconstrained NPMLE and the proposed estimator of Sect. 2 that assumes constant misclassification rates at all times (i.e. assumes *A* = ∞). For non-extreme values of *A*, these two estimators were compared to determine which approach would be most accurate if it is suspected that the data is misclassified within a specific window and not misclassified otherwise. Each simulation consisted of 1000 data sets.

Table 2 shows the results of both estimators of *F* at a selection of monitoring times, chosen systematically to depict the overall spread. These monitoring times are evaluated assuming windows of length *A* = 0 and *A* = ∞. The results are as expected where the NPMLE of no misclassification performs best for a window of *A* = 0 (where no individuals are subject to misclassification) and the proposed NPMLE, adjusted for constant misclassification, performs best for a window of *A* = ∞ (where all individuals are subject to misclassification with approximately 20% misclassified). Table 3 provides similar results where the window length varies, allowing approximately 60% and 82% of individuals to be subject to misclassification, the actual average percent misclassified also being indicated in the table. The results of Table 3 are perhaps not as expected where the adjusted NPMLE only outperforms the unconstrained NPMLE when a very high proportion of individuals are subject to misclassification. Even when 82% are subject to misclassification, evidence in favor of the adjusted NPMLE is not overwhelming.

In practice, an investigator necessarily does not know the underlying *F* and so cannot immediately assess which approximate NPMLE to use, the one that assumes no misclassification or the one that assumes a constant rate of misclassification over time. In this situation, it is possible however to carry out a simulation using either estimator as the assumed ‘true’ *F* to examine performance. We examine this further in the next simulation with an additional wrinkle to the misclassification model in the non-extreme simulations.

If there is misclassification due to laboratory error in the (current status) screening instrument, all individuals will be subject to this error. However, even with constant laboratory misclassification, there may also be increased (and potentially asymmetric) misclassification rates close to the true failure event. Table 4 presents results of simulations from the HPV data where the true underlying distribution is assumed to be the unconstrained NPMLE as obtained through the standard pool-adjacent-violators algorithm. A constant laboratory error is assumed, giving classification rates of *α* = 0.8 and *β* = 0.9 outside the window and *α* = 0.73 and *β* = 0.9 within the window, indicating an additional deterioration in sensitivity close to the underlying failure time. In computing the constant misclassification adjusted NPMLE the values *α* = 0.73 and *β* = 0.9 were assumed.

In the simulations for the HPV data it must be noted that unlike the simulations in Tables 2 and 3, when *A* = 0 there is still misclassification present, at a constant rate of *α* = 0.8*, β* = 0.9. This explains the lack of accuracy in the unconstrained NPMLE for *A* = 0 which assumes no misclassification (and similarly for the constant misclassification adjusted NPMLE which uses the incorrect misclassification probabilities). When *A* = ∞ the results are as expected with the adjusted NPMLE more favorable as in this instance there is constant misclassification at rates *α* = 0.73*, β* = 0.9. Under the intermediate situations, with complex window misclassifications and non-zero and finite values for *A*, the simulations suggest that there is a slight preference for the adjusted NPMLE in terms of bias although there is a small price to be paid for additional variability. Mean squared error gives the nod here to the unconstrained NPMLE at least with these two possibilities for the window parameter *A*.

In either case, the simulations suggest a way to remove the bias for either estimator when *A* is finite and non-zero. The bias-adjusted algorithm is as follows: (i) compute a suitable simulation ‘guess’ for the *F* to be used in the simulations; (ii) simulate data assuming this ‘guess’ is the truth, with the assumed value for *A* and the relevant misclassification probabilities within and without the window defined by *A*; (iii) estimate the bias at all values of *C* of interest by comparing the simulation average with either of the original estimators; (iv) remove this estimated bias from the original estimator. Either the unconstrained NPMLE or the constant misclassification adjusted NPMLE could be used for the ‘guess’, although we prefer to hedge our bets by using the average of these two straightforward estimators since the simulations seem to suggest that the bias for the two estimators is sometimes in opposite directions, particularly in the tails where the biases tend to be most severe. Note that this algorithm can be used for more complex misclassification models that might be anticipated.

To formalize the above steps of the bias adjustment approach, note that the bias in the unconstrained NPMLE at *t*_0_ is $bias_{0}\left(t_{0}\right)=E\left({\hat{F}}_{0}\left(t_{0},F\right)\right)-F\left(t_{0}\right)$, where *F* is the assumed true data generating distribution, and ${\hat{F}}_{0}$ is the unconstrained NPMLE. We estimate the bias by substituting ${\hat{F}}_{g}$ for *F* in each of the terms in *bias*_0_(*t*_0_) and estimate the expectation through simulations, thus yielding $b\hat{ia}s_{0}\left(t_{0}\right)=\hat{E}\left({\hat{F}}_{0}\left(t_{0},{\hat{F}}_{g}\right)\right)-{\hat{F}}_{g}\left(t_{0}\right)$. This estimate, ${\hat{F}}_{g}$, could be the unconstrained estimate, $\hat{F}\left(t_{0},F\right)={\hat{F}}_{0}\left(t_{0},F\right)$, the estimate under constant misclassification, $\hat{F}\left(t_{0},F\right)={\hat{F}}_{\infty }\left(t_{0},F\right)$, or the average of both estimates, $\hat{F}\left(t_{0},F\right)=\left({\hat{F}}_{0}\left(t_{0},F\right)+{\hat{F}}_{\infty }\left(t_{0},F\right)\right)/2$. Finally we produce the bias-adjusted estimate by ${\hat{F}}_{0}^{ba}\left(t_{0}\right)={\hat{F}}_{0}\left(t_{0},F\right)-b\hat{ia}s_{0}\left(t_{0}\right)$. Similarly, for the constant misclassified adjusted NPMLE, $b\hat{ia}s_{\infty }\left(t_{0}\right)=\hat{E}\left({\hat{F}}_{\infty }\left(t_{0},{\hat{F}}_{g}\right)\right)-{\hat{F}}_{g}\left(t_{0}\right)$ where ${\hat{F}}_{g}$ is chosen as before; this estimated bias can then be used to ‘correct’ ${\hat{F}}_{\infty }$ as before.

Tables 3 and 4 provide the simulated performance of these bias adjusted versions of the original estimators for the same simulations considered before. In constructing the bias adjusted estimators, a sample size of 500 was used in step (ii) of the algorithm above and 1,000 simulations of step (ii) were carried out. It is clear from the results reported in Tables 3 and 4 that the bias adjusted estimators have significantly improved performance in terms of bias with only modest increases in variability. The improvement is more noticeable in Table 3 as the original bias is much greater. Note that bias adjustments can also be calculated when *A* = 0 but are not presented in the table.

### 2.5 Regression models

We briefly consider the extension of the above ideas to the regression context where interest focuses on the effects of a (potentially multidimensional) covariate *X*. Much of the literature on current status data has exploited the correspondence between standard regression models for the underlying failure time and generalized linear models for the observed current status outcome in both the parametric and semiparametric setting. These ideas are reviewed in Jewell and van der Laan (2004) and extended to more complex failure time data in Jewell (2007).

To adapt these techniques to accommodate misclassification we use the ideas of binary generalized linear models with outcomes subject to misclassification (Neuhaus 1999). Focusing on the constant misclassification model, and with the same assumptions as before, it follows that

P(Δ=1∣X,C)=(α+β−1)P(Y=1∣X,C)+(1−β)

and so

$$P(\Delta =1|X,C)=(\alpha +\beta -1)g^{-1}\left(\eta_{x,c}\right)+(1-\beta ),$$

where *g* is the link function in the induced generalized linear model for *Y*. In addition, in most models, the regression term *η*_*x,c*_ is also additive in *x* and *c*. It follows that the observed outcome Δ also follows a generalized linear model with a modified link function, namely;

$$g^{*}(P(\Delta =1|X,C))=g\left\{\frac{P(\Delta =1|X,C)-(1-\beta )}{\left(\alpha +\beta -1\right)}\right\}.$$

For example, assuming a Weibull regression model for *T*, the generalized linear model for *Y* in *X* and *C* involves *g*, the complementary log–log link function. We fit regression models to the HPV data (a) assuming no errors in the response variable (therefore using *g* directly), and (b) adjusting for errors with constant classification rates *α* = 0.73 and *β* = 0.9 (using g*). These assumed classification rates allow both for laboratory error and the possibility that some negative tests fail to detect prior HPV infection as discussed in Sect. 2.2. Note that the parameter estimates in both models have proportional hazards interpretations on age at first infection with HPV, according to the Weibull regression model assumption for *T*, as distinct from the simple cross-sectional interpretations discussed in Neuhaus (1999). The results of both models are presented in Table 5, along with the observed ratio of parameter estimates. The generalized linear model induced by Weibull regression indicates that age at screening must be included in the model additively on the log scale. The standard errors were obtained from the observed information matrix and were calculated using PROC GENMOD in SAS version 9.1.

According to models (a) and (b), respectively, the hazard of first HPV infection are increased by 6 and 11% for those who currently smoke (Smoke now = 1) to those who do not smoke (Smoke now = 0), holding other covariates in the model fixed; clearly this effect is not significant. On the other hand, the hazard of HPV infection is reduced by 38 and 50% for those who have had any other prior sexually transmitted disease (STD = 1) compared with those who have not (STD = 0); this effect is quite strikingly significant, at least when misclassification is accounted for. As reported by Neuhaus (1999), the ratio of the parameter estimates suggest that ignoring the errors in the HPV screening test leads to substantially biased estimates of the associations of covariates with infection status, with the direction of the bias reflecting attenuation towards the null. Our findings are qualitatively similar to those of Neuhaus (1999) although we show a somewhat lower effect for prior STDs, presumably due to our allowance for additional error.

## 3 Discussion

We have discussed the NPMLE of a distribution function based on current status data subject to misclassification. The ideas are also easily extended to regression models for the underlying survival time. We have illustrated the latter using a parametric regression model. Alternative methods to allow for misclassification in the current status response include the simulation extrapolation (SIMEX) method (for the regression setting, see Hardin et al. 2003, for the SIMEX method applied to standard generalized linear models). Recently, Küchenhoff et al. (2006) applied SIMEX to binary outcome data associated with a generalized linear model and compared results to the maximum likelihood approach espoused by Neuhaus (1999).

Although we considered a parametric regression model, semi-parametric survival models can also be analyzed using the ideas of Shiboski (1998) on semi-parametric generalized additive models. In this case, the technique of adjusting the link function to allow for misclassification, discussed in Sect. 2.5, can also be used. SIMEX provides an alternative approach. In addition, the bias adjustment algorithm discussed in Sect. 2.4 can also be applied in the regression context, in particular to allow for more complex misclassification models.

Throughout we have assumed that the misclassification rates and window of misclassification, if appropriate, are known exactly. In some cases, the rates may have to be estimated from a validation sample where the true response is measurable perhaps by use of an expensive ‘gold standard’ technique. This data can then be incorporated into a full likelihood that will then account for the uncertainty in estimation of the misclassification rates. In principal, a similar approach could be used for validation data that provided information on the value of *A* or the size of the misclassification window. However, estimation of the value of *A* is itself a much studied non-trivial estimation problem in detecting the time of transition of binomial classification rates. We leave these interesting extensions to future work.

## Figures and Tables

**Table 1:** Confidence interval estimation for the adjusted (*α* = 0.73*, β* = 0.9) NPMLE at three monitoring times obtained using the *m* out of *n* bootstrap for various values of *m* ranging from 9 to 423

*t*_0_	15.3 years	19 years	22 years
$\hat{F}\left(t_{0}\right)$	0.609	0.763	0.793
*m* = 9 (*n*^1/3^)	[0.471 0.747]	[0.614 0.912]	[0.718 0.868]
*m* = 29 (*n*^1/2^)	[0.407 0.811]	[0.646 0.880]	[0.718 0.868]
*m* = 88 (*n*^2/3^)	[0.311 0.907]	[0.646 0.880]	[0.687 0.899]
*m* = 154 (*n*^3/4^)	[0.396 0.822]	[0.667 0.859]	[0.665 0.921]
*m* = 216 (*n*^0.8^)	[0.407 0.811]	[0.667 0.859]	[0.655 0.931]
*m* = 423 (*n*^0.9^)	[0.449 0.769]	[0.688 0.838]	[0.676 0.910]

**Table 2:** Simulation averages (standard deviations) of two estimators of the distribution function *F* (Exponential with mean 2) at 5 monitoring times, when the data generating distribution is either subject to always being misclassified (*A* = ∞), or never being misclassified (*A* = 0)

*C*	0.4	0.8	1.4	1.8	2.8
	*F*(*C*) = 0.181	*F*(*C*) = 0.330	*F*(*C*) = 0.503	*F*(*C*) = 0.593	*F*(*C*) = 0.753
*A* = 0					
0% (0)%					
NPMLE_0_	0.178(0.055)	0.331(0.063)	0.496(0.059)	0.591(0.056)	0.760(0.049)
NPMLE_∞_	0.022(0.043)	0.218(0.104)	0.494(0.098)	0.652(0.094)	0.923(0.068)
*A* = ∞					
100% (20)%					
ZNPMLE_0_	0.306(0.056)	0.397(0.056)	0.500(0.051)	0.557(0.047)	0.662(0.050)
NPMLE_∞_	0.178(0.091)	0.329(0.094)	0.500(0.086)	0.593(0.078)	0.769(0.084)

The resulting % subject to misclassification (average % actually misclassified) are also given for each simulation. NPMLE_0_ and NPMLE_∞_ represent the unconstrained NPMLE and the NPMLE adjusted for constant response misclassification, respectively

**Table 3:** Simulation averages (standard deviations) of two estimators of the distribution function *F* (Exponential with mean 2) at 5 monitoring times when the data generating distribution is subject to constant misclassification (*α* = 0.8*, β* = 0.8) only within a window of length 2*A* around the underlying failure time

*C*	0.4	0.8	1.4	1.8	2.8
	*F*(*C*) = 0.181	*F*(*C*) = 0.330	*F*(*C*) = 0.503	*F*(*C*) = 0.593	*F*(*C*) = 0.753
*A* = 1.5					
60% (12%)					
NPMLE_0_	0.225(0.057)	0.327(0.057)	0.454(0.056)	0.543(0.057)	0.732(0.052)
NPMLE_∞_	0.063(0.069)	0.214(0.095)	0.424(0.094)	0.571(0.095)	0.884(0.079)
Bias adjusted					
NPMLE_0_	0.177(0.088)	0.315(0.097)	0.467(0.101)	0.575(0.102)	0.775(0.084)
NPMLE_∞_	0.172(0.103)	0.337(0.116)	0.485(0.118)	0.588(0.119)	0.769(0.104)
*A* = 2.5					
82% (16%)					
NPMLE_0_	0.258(0.055)	0.358(0.056)	0.470(0.056)	0.530(0.057)	0.673(0.051)
NPMLE_∞_	0.104(0.084)	0.263(0.095)	0.451(0.087)	0.549(0.085)	0.787(0.090)
Bias adjusted					
NPMLE_0_	0.185(0.093)	0.320(0.099)	0.487(0.092)	0.551(0.090)	0.729(0.094)
NPMLE_∞_	0.184(0.109)	0.331(0.117)	0.506(0.107)	0.559(0.105)	0.731(0.109)

Window lengths of *A* = 1.5 and *A* = 2.5 are evaluated. The resulting % subject to misclassification (average % misclassified) are also given for each simulation. NPMLE_0_ and NPMLE_∞_ represent the unconstrained NPMLE and the NPMLE adjusted for constant response misclassification, respectively. The corresponding bias adjusted estimates (standard deviations) for each estimator under the different window lengths are also presented

**Table 4:** Simulation averages (standard deviations) of two estimators of the distribution function *F* (unconstrained NPMLE from the HPV data) at 5 monitoring times when the data generating distribution is subject to misclassification that varies with time

*C*	15 years	16.2 years	19.2 years	21.7 years	23.2 years
	*F*(*C*) = 0.484	*F*(*C*) = 0.539	*F*(*C*) = 0.581	*F*(*C*) = 0.600	*F*(*C*) = 0.661
*A* = 0					
0% (0%)					
NPMLE_0_	0.414(0.086)	0.464(0.054)	0.511(0.031)	0.540(0.039)	0.584(0.069)
NPMLE_∞_	0.498(0.137)	0.578(0.085)	0.652(0.049)	0.698(0.061)	0.766(0.102)
*A* = 4.5					
43% (7%)					
NPMLE_0_	0.388(0.084)	0.433(0.057)	0.498(0.034)	0.532(0.042)	0.584(0.076)
NPMLE_∞_	0.457(0.132)	0.528(0.090)	0.632(0.054)	0.686(0.067)	0.764(0.111)
Bias adjusted					
NPMLE_0_	0.435(0.153)	0.475(0.113)	0.537(0.100)	0.562(0.115)	0.612(0.168)
NPMLE_∞_	0.475(0.187)	0.472(0.149)	0.524(0.141)	0.544(0.147)	0.606(0.191)
*A* = 8					
86% (15%)					
NPMLE_0_	0.383(0.081)	0.426(0.054)	0.474(0.030)	0.513(0.045)	0.573(0.078)
NPMLE_∞_	0.449(0.129)	0.517(0.086)	0.594(0.047)	0.655(0.071)	0.747(0.114)
Bias adjusted					
NPMLE_0_	0.437(0.153)	0.473(0.113)	0.520(0.090)	0.553(0.114)	0.610(0.172)
NPMLE_∞_	0.473(0.189)	0.472(0.150)	0.513(0.132)	0.542(0.154)	0.604(0.203)
*A* = ∞					
100% (20%)					
NPMLE_0_	0.384(0.083)	0.428(0.052)	0.472(0.032)	0.496(0.038)	0.544(0.075)
NPMLE_∞_	0.451(0.129)	0.521(0.083)	0.590(0.050)	0.629(0.060)	0.702(0.111)

Classification rates of *α* = 0.8 and *β* = 0.9 are assumed outside the window and rates of *α* = 0.73 and *β* = 0.9 are assumed within a window of length 2*A* around the underlying failure time. Window lengths of *A* = 0, 4.5, 8, ∞ are evaluated. The resulting % subject to misclassification (average % misclassified) are also given for each simulation. NPMLE_0_ and NPMLE_∞_ represent the unconstrained NPMLE and the NPMLE adjusted for constant (*α* = 0.73, *β* = 0.9) misclassification, respectively. The corresponding bias adjusted estimates (standard deviations) for each estimator in the windows of length *A* = 4.5 and *A* = 8 are also presented

**Table 5:** Estimates (and standard errors) of the log Relative Hazard (RH) for time to first HPV infection, which is assumed to follow a Weibull distribution

Covariate	Log (RH): Model (a) $\left({\hat{\beta }}^{*}\right)$ Ignoring misclassification	Log(RH): Model (b) $\left(\hat{\beta }\right)$ Adjusted for misclassification	${\hat{\beta }}^{*}/\hat{\beta }$
Smoke now	0.056(0.108)	0.103(0.144)	0.544
STD	−0.479(0.299)	−0.698(0.258)	0.686
Log(age at screening)	0.822(0.455)	1.269(0.552)	0.648

Model (a) ignores misclassification in the response variable and Model (b) incorporates constant misclassification corresponding to *α* = 0.73 and *β* = 0.9
